# IMU Data and GPS Position Information Direct Fusion Based on LSTM

**DOI:** 10.3390/s21072500

**Published:** 2021-04-03

**Authors:** Xingxing Guang, Yanbin Gao, Pan Liu, Guangchun Li

**Affiliations:** 1 College of Intelligent System Science and Engineering, Harbin Engineering University, Harbin 150001, China; gaoyanbin@hrbeu.edu.cn (Y.G.); lgc_67@hrbeu.edu.cn (G.L.); 2 Beijing Institute of Control and Electronic Technology, Beijing 100032, China; liupan003@hrbeu.edu.cn

**Keywords:** long short-term memory, IMU data, hyperparameter evaluation, positioning

## Abstract

In recent years, the application of deep learning to the inertial navigation field has brought new vitality to inertial navigation technology. In this study, we propose a method using long short-term memory (LSTM) to estimate position information based on inertial measurement unit (IMU) data and Global Positioning System (GPS) position information. Simulations and experiments show the practicability of the proposed method in both static and dynamic cases. In static cases, vehicle stop data are simulated or recorded. In dynamic cases, uniform rectilinear motion data are simulated or recorded. The value range of LSTM hyperparameters is explored through both static and dynamic simulations. The simulations and experiments results are compared with the strapdown inertial navigation system (SINS)/GPS integrated navigation system based on kalman filter (KF). In a simulation, the LSTM method’s computed position error Standard Deviation (STD) was 52.38% of what the SINS computed. The biggest simulation radial error estimated by the LSTM method was 0.57 m. In experiments, the LSTM method computed a position error STD of 23.08% using only SINSs. The biggest experimental radial error the LSTM method estimated was 1.31 m. The position estimated by the LSTM fusion method has no cumulative divergence error compared to SINS (computed). All in all, the trained LSTM is a dependable fusion method for combining IMU data and GPS position information to estimate position.

## 1. Introduction

INS is one of the important means for realizing vehicle positioning. It uses dead reckoning technology, based on IMU data, to compute the vehicle position information. SINS functions are a group of nonlinear equations. The position error, calculated based on inertial sensor data, includes two aspects, sensor measurement error and calculation error. When the IMU is confirmed, the position calculation method will directly affect the position estimation error. The nonlinear SINS functions should not cause divergent position error [1,2,3].

Today, deep learning is widely used in various fields, such as image processing, semantic understanding, speech processing, and data processing [4,5,6,7,8,9,10]. In recent years, deep learning has also been popular in the inertial navigation field [11]. Deep learning methods, like LSTM network and recurrent neural networks (RNN), have many advantages over forward networks for nonlinear system modeling [12,13]. The nonlinear autoregressive neural network is combined with an Unscented Kalman Filter (UKF) with external inputs to improve the position and velocity precision of the INS/Global Navigation Satellite System (GNSS) during GNSS outages [14]. RNN is used to improve the UKF for estimating and compensating the random drift of inertial sensors in real time [15]. To overcome the problem caused by overrange inertial measurement data, the faster region-based convolutional neural network (RCNN) method is proposed to realize robust autonomous navigation [16]. To improve the navigation ability of inertial/GPS integrated navigation system, LSTM is used to improve the system’s error prediction ability [17]. LSTM is used to estimate the com-cop inclination angle during walking based on inertial sensors [18]. LSTM-RNN is also proposed to denoise the MEMS IMU output signals [19]. A number of studies have shown that the application of deep learning to the inertial navigation field brings about new vitality to inertial technology [20,21,22].

In this study, a method is proposed that uses LSTM to estimate the position information based on the IMU data and GPS position information. Aiming at the structure of LSTM, the value range of LSTM hyperparameters is explored through the simulation. The results of LSTM estimation are compared with those computed with only SINS and those computed with a SINS/GPS loosely coupled KF during static positioning and dynamic positioning.

## 2. LSTM Net

LSTM takes sequences of information and uses recurrent mechanisms and gate techniques [23]. The structure, training/testing, and hyperparameters of LSTM are described in this section.

### 2.1. LSTM Net Structure

**Figure 1:**
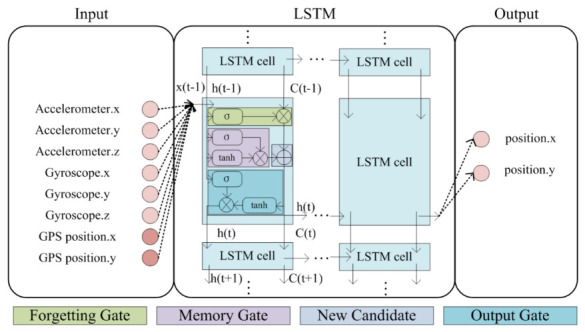
The LSTM net structure of inertial position estimation.

As shown in Figure 1, the rounded rectangle in the LSTM cell is the neural network layer of the LSTM [18,24]. The combination of a rounded rectangle and a multiplication operation is a gate. Gates are used to control the state of the LSTM cell. The repeating module in an LSTM cell contains four interacting layers: a forgetting gate layer, an input gate layer, a new candidate layer, and an output gate layer. In the LSTM net structure, the gate is realized by a sigmoid function:

(1) $$\sigma \left(x\right)=1/1+e^{-x}.$$

- The forgetting gate layer is made by a sigmoid layer [25]:
  (2) $$\;f\left(t\right)=\sigma \left(W_{f}\left[h\left(t-1\right),x\left(t\right)\right]+b_{f}\right).$$
- The input gate layer decides what new information is going to be stored in the cell state. It has two steps. First, a sigmoid layer decides which values are to be updated. Next, a hyperbolic tangent function (tanh) layer creates a vector of new candidate values, $\tilde{C}\left(t\right)$, that could be added to the state:
  (3) $$i\left(t\right)=\sigma \left(W_{i}\left[h\left(t-1\right),x\left(t\right)\right]+b_{i}\right)$$
  (4) $$\tilde{C}\left(t\right)=\tan h\left(W_{C}\left[h\left(t-1\right),x\left(t\right)\right]+b_{C}\right).$$
- The new candidate layer is used to update the old cell state, $C\left(t-1\right)$, into the new cell state $C\left(t\right)$, as in the below equation:
  (5) $$C\left(t\right)=f\left(t\right)\times C\left(t-1\right)+i_{i}\times \tilde{C}\left(t\right).$$
- The output gate layer: the output is decided in this layer. First, it is going to be forgotten that the net decided to forget earlier. Then, we scale the new candidate values. Finally, we decide the output. The output is based on the cell states. The output computation is as in the below equations:
  (6) $$O\left(t\right)=\sigma \left(W_{O}\left[h\left(t-1\right),x\left(t\right)\right]+b_{O}\right)$$
  (7) $$h\left(t\right)=O\left(t\right)\times \tanh\left(C\left(t\right)\right).$$

As to the LSTM structure, the LSTM can use $i\left(t\right)$ to decide when to keep or override information in memory cell $C\left(t\right)$, and $O\left(t\right)$ to decide when to access memory cell $C\left(t\right)$ and when to prevent other units from being perturbed by $C\left(t\right)$. The $O\left(t\right)$ is the output information and the $h\left(t\right)$ is the output about the LSTM structure.

Due to the forgetting gate in the structure, the LSTM net can realize the conditional prediction based on time. In another words, during the learning of a large amount of IMU data and GPS position data, the LSTM learns the position information characteristics related to time. The SINS computes the current inertial position information based on the last step. The SINS/GPS integrated navigation system using KF to estimate the position information is also based on the last step estimation at the current moment. Those processed can be regarded as a conditional prediction based on time. Therefore, we can use LSTM to estimate the position information based on IMU data and GPS position information. This means that the position information is defined as the features of IMU data. To learn the position features from IMU data, we also input the GPS position data to the LSTM net as the target.

### 2.2. LSTM Training and Testing Settings

**Figure 2:**
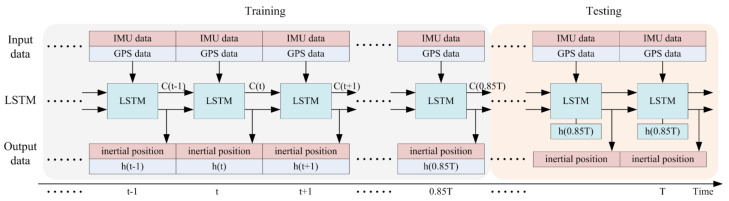
Training and testing the LSTM to estimate position information.

As shown in Figure 2, in this study, the training rate is 0.85. This means that 85% of the input dataset is the training set and the other 15% is the test set. Input and output size are based on the practical application of the LSTM.

As shown in Figure 1 and Figure 2, the input set includes two parts: IMU data, including three dimensions of gyroscope measurement data and three dimensions of accelerometer measurement data; and GPS data, including two dimensions of position information. The IMU data are the input of the LSTM, and the GPS data are the training target data. The output dataset includes two parts: the predicted inertial position, in two dimensions, and the output of the LSTM, with the number of dimensions based on the LSTM structure.

In LSTM, the purpose of net training is to optimize the weights and biases. The state activation function is the tanh, while the optimizer function is the adaptive moment estimation (Adam) stochastic gradient descent method with high convergence performance [18,26,27].

We use the root mean stand error (RMSE) to describe the training cost:

(8) $$RMSE=\sqrt{\frac{1}{n}\sum_{i=1}^{n}{\left(y_{pi}-y_{i}\right)}^{2}},$$

where, $y_{pi}$ is the target GPS position data and$\;y_{i}$ is the inertial position predicted by LSTM.

### 2.3. Hyperparameters of LSTM

Hyperparameters need to be adjusted during LSTM training to make sure of the training cost in a confidence interval, including the time step, cell size, batch size, learning rate, and forgetting rate.

- Time step: in LSTM, the purpose of net training is to optimize the weights and biases, to make sure the output errors are within a certain range. It is worth noting that the calculation of weights and biases is independent of the time step. In other words, the time step of LSTM can be set to any number. In this study, we expect that when the input IMU and target GPS data are at the same frequency, each frame of IMU data can be calculated to obtain the position information. Therefore, the time step in this study is 1.
- Cell size: the hidden layer unit size, also called the hidden size. Cell size defines the deep degree of LSTM.
- Batch size: also called mini-batch size. In the mini-batch gradient descent method, the data are divided into several batches and the parameters are updated according to the batches. In this way, the data in a batch jointly determine the direction of the gradient, so that the descent is not easy to determine, and the randomness is reduced. On the other hand, because the sample size of the batch is much smaller than the whole dataset, the computation burden will be reduced.
- The learning rate and forgetting rate control the speed of adjusting weights and biases of neural network based on the loss gradient.

## 3. Simulation

To select appropriate hyperparameters such as the batch size, cell size, learning rate, and forgetting rate, we performed many simulations with different hyperparameters in this section.

### 3.1. Hyperparameter Evaluation

There are two simulation cases, static position estimation and dynamic position estimation. The IMU data used in the simulation are generated by the inertial simulator. The latitude and longitude of the initial location are set to 35° N and 108° E, respectively. The gyro constant drifts and random drifts are set to 0.02°/h and 0.001°/h, respectively. The accelerometer’s constant bias and random bias are 1 × 10^−4^ g and 1 × 10^−5^ g, respectively, where g is the local gravitation. In the static case, the simulation time is 60 s. In the dynamic case, the simulation time is 780 s, and the velocity is ${\left[1,1,0\right]}^{T}$ m/s. For each hyperparameter set, there are 100 training and testing events based on the simulated IMU data. We performed statistical tests on the hyperparameters used to train the LSTM to estimate the location based on the IMU data and GPS position information. The hyperparameters in the evaluation distribution point are the set that never cause overfitting or underfitting when training the LSTM to estimate the inertial position. They are recoded as shown in the following figures.

**Figure 3:**
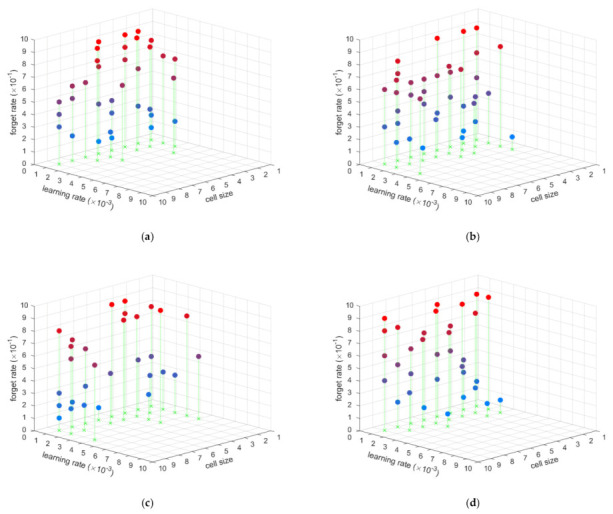
The hyperparameter evaluation for static position estimation: (**a**) batch size is 5, (**b**) batch size is 10, (**c**) batch size is 15, (**d**) batch size is 20.

**Figure 4:**
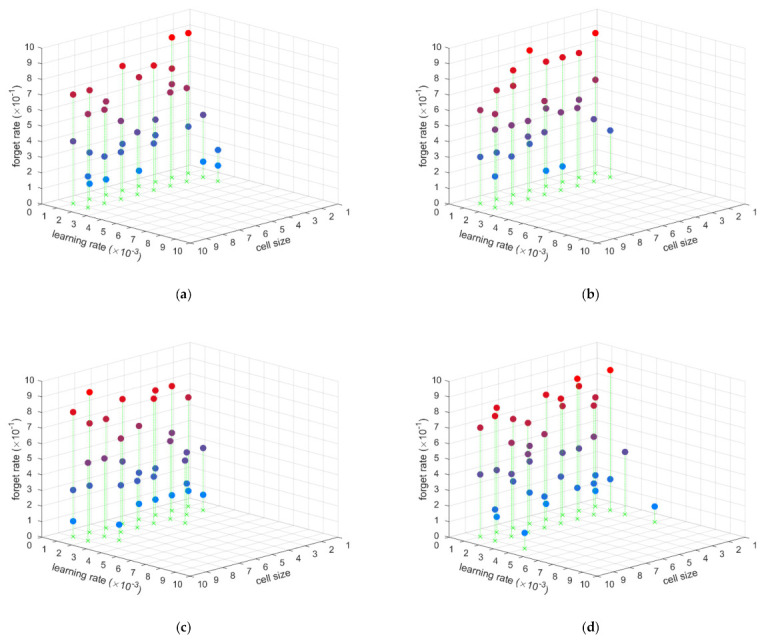
The hyperparameter evaluation for dynamic position estimation: (**a**) batch size is 5, (**b**) batch size is 10, (**c**) batch size is 15, (**d**) batch size is 20.

As each subgraph shows in Figure 3 and Figure 4, the hyperparameter evaluation distribution is similar when the batch size is different, and the hyperparameter evaluation distribution is also similar with different cell sizes and forgetting rates. However, different learning rates obviously affected the hyperparameter evaluation. Thus, the forgetting rate, cell size, and batch size have little influence on the LSTM used to estimate the position based IMU data. The important hyperparameter is the learning rate. From those simulations, when the learning rate is between 0.001 and 0.002, the LSTM is able to estimate the position based IMU data well. This hyperparameter evaluation applies to both static and dynamic situations. In another words, the static position estimation LSTM net and dynamic position estimation LSTM net have the same hyperparameter range. This means that an LSTM net with the same hyperparameters can be trained to estimate position based IMU data, no matter whether the conditions are static or dynamic.

### 3.2. Position Estimation

In this section, the position information estimated by LSTM and the position information obtained by the inertial system functions are compared in static conditions and moving conditions. The uniform rectilinear motion data are simulated in the dynamic case.

**Figure 5:**
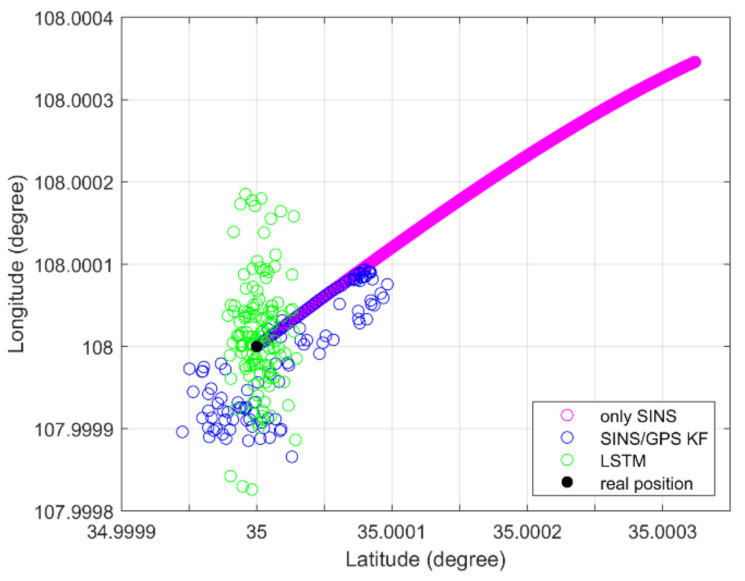
Static position information of simulation.

As shown in Figure 5, an LSTM net can fuse the IMU and GPS data to estimate the position information, as with the SINS/GPS loosely coupled based KF, in static simulation. Both an LSTM net and a loosely coupled KF-based system can restrain the divergence of only SINS. The position error STDs of only SINS, KF, and LSTM are 1.47 m, 0.88 m, and 0.77 m, respectively. The KF method computed position error STD is 59.86% for only SINSs, and the LSTM method estimated position error STD is 52.38% for only SINSs. The LSTM estimation position is more convergent than the KF method estimated.

The position error in the dynamic case is described by radial error. The radial error is defined by

(9) $$e_{radial}=\sqrt{{\left(\left(L_{e}-L_{r}\right)R_{N}\right)}^{2}+{\left(\left(\lambda_{e}-\lambda_{r}\right)R_{M}\cos L_{r}\right)}^{2}},$$

where $L_{e}\;$and $\lambda_{e}$ are the estimation latitude and estimation longitude, respectively, and $L_{r}\;$and $\lambda_{r}$ are the reference latitude and reference longitude, respectively. $R_{N}=R_{e}/\sqrt{1-e^{2}\sin^{2}L}$, $R_{M}=R_{e}\left(1-e^{2}\right)/{\left(1-e^{2}\sin^{2}L\right)}^{3/2}$, $R_{e}$ is the ellipse major axis, and e is the ellipse eccentricity.

**Figure 6:**
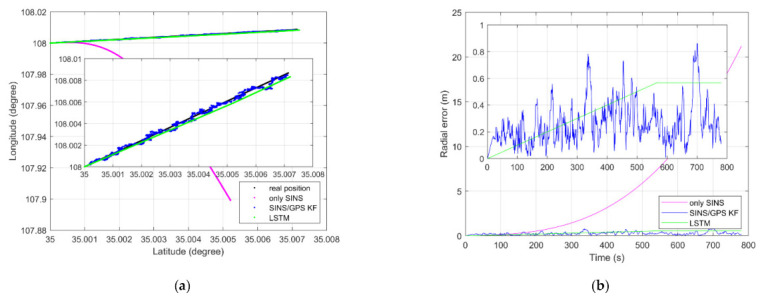
(**a**) Dynamic position estimation of simulation; (**b**) dynamic position radial error of simulation.

As shown in Figure 6a, LSTM net can fuse the IMU and GPS data to estimate the position information, like the SINS/GPS loosely coupled KF-based system, in a dynamic simulation. Both the LSTM net and the loosely coupled KF-based system can restrain the divergence of only SINS. As shown in Figure 6b, the biggest radial error of only SINS, KF method, and LSTM method are 21.21 m, 0.86 m, and 0.57 m, respectively. As Figure 6a,b shows, the position information obtained by only SINS has a nonlinear divergence error. The radial error of only SINS will continue to grow over time. However, the position error of KF and LSTM does not increase over time. The radial error of KF oscillates around 0. The radial error of LSTM is piecewise linear. Before 580 s, the position information estimated by LSTM has a linear divergence error. After 580 s, it becomes a constant error. LSTM can enforce constant error through “constant error carousels” within the forgetting gate during enough training steps [23]. The radial error of the LSTM estimation position accords with this conclusion.

As the simulation results show, the LSTM net can fuse the IMU and GPS data to estimate the position information and restrain the divergence error of only SINS, as with the SINS/GPS loosely coupled KF-based system.

## 4. Experiments

**Figure 7:**
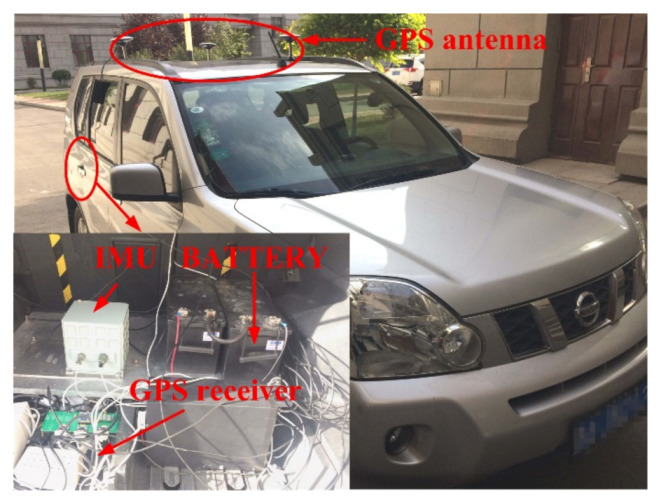
The experimental equipment.

An experimental system was assembled to evaluate the proposed approach, as shown in Figure 7. The experimental equipment included an IMU, a GPS, and a power system on the vehicle. The IMU constituted a three-axis fiber-optic gyroscope with three accelerometers on each gyro-axis. The GPS was UR370 form UNICORE. The IMU, GPS receiver, and power system are in the vehicle trunk. The IMU is fixed on the vehicle via a steel plate that is parallel to the under panel of the vehicle. During the experiment, the IMU and GPS data were recoded. Both IMU data and GPS data included the GPS time. In this study, the GPS provided the position information target. The GPS data and IMU data were synchronized by their GPS times. There were two cases in the experiment, one static and the other dynamic. In static conditions, the vehicle stops 2 min. In dynamic conditions, the vehicle was driving in a uniform rectilinear motion along the road. The driving distance was 450 m and the driving speed was 2 km/s.

**Figure 8:**
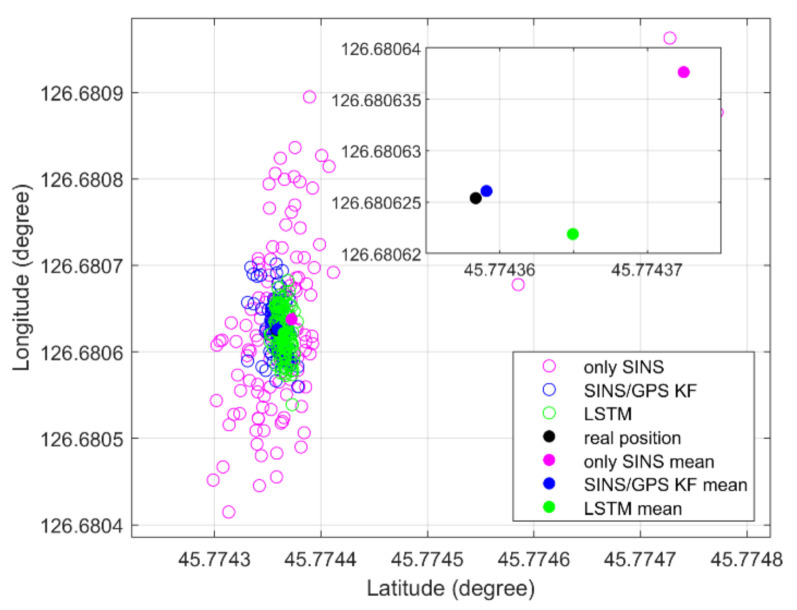
Static position of experiment.

As shown in Figure 8, an LSTM net can fuse the IMU and GPS data to estimate the position information, like the SINS/GPS loosely coupled KF-based system, in a static experiment. Both the LSTM net and loosely coupled KF-based system can restrain the divergence of only SINS. The position error STDs of only SINS, KF method, and LSTM method are 1.30 m, 0.33 m, and 0.30 m, respectively. The KF method computed position error STD is 25.38% for only SINSs, and the LSTM method computed position error STD is 23.08% for only SINSs. The LSTM estimation position is more convergent than the KF method estimated.

**Figure 9:**
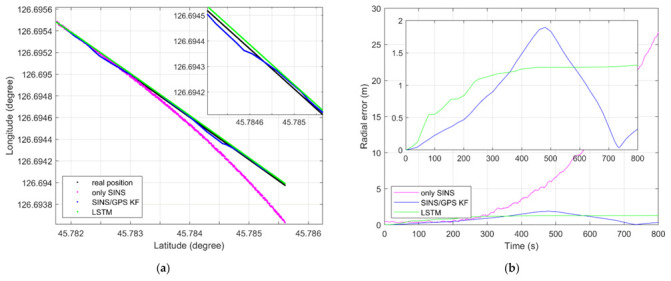
(**a**) Dynamic position estimation of experiment; (**b**) dynamic position radial error of experiment.

As shown in Figure 9a, an LSTM net can fuse the IMU data and GPS position information to estimate the position information, like the SINS/GPS loosely coupled KF-based system, in a dynamic experiment. Both the LSTM net and loosely coupled based KF can restrain the divergence of only SINS. As shown in Figure 9b, the largest radial error of only SINS, KF method, and LSTM method are 26.71 m, 1.89 m, and 1.31 m, respectively. As Figure 6a,b shows, the position information obtained by only SINS has nonlinear divergence error. The radial error of only SINS will continue to grow over time. However, the position error of KF method and LSTM method does not increase over time. The radial error of the KF method oscillates around 0. The radial error of LSTM method is piecewise linear. Before 450 s, the position information estimated by LSTM has linear divergence error. After 450 s, it becomes constant error. LSTM can enforce constant error through “constant error carousels” within the forgetting gate during enough training steps [23]. The radial error of the LSTM estimation position accords with this conclusion.

As the experimental results show, the LSTM net can fuse the IMU and GPS data to estimate the position information and restrain the divergence error of only SINS, like the SINS/GPS loosely coupled KF-based system.

## 5. Discussion

As the results of the simulations and experiments have shown, the effectiveness of the proposed method, using an LSTM net to fuse the IMU data and GPS to estimate the position information, has been proven in both static and dynamic conditions. Using the LSTM net to fuse IMU data and GPS position information can restrain the divergence of only SINS, like the SINS/GPS loosely coupled KF-based navigation system can.

The position error of only SINS includes sensor measurement error and calculation error. In this study, the simulation and experiment are based on the same IMU data and the same GPS position information. Thus, different position errors were found based on the calculation methods.

The model of position information calculation determines the character of the computational error. For the only SINS, as Equations (A3) and (A6) in Appendix A show, the position information and position error of only SINS are related to the inertial velocity computation and the inertial velocity error. The velocity information is integrated by geometric acceleration, and we get the position information by integrating the velocity information. The SINS functions and SINS error functions are nonlinear. The nonlinear error will accelerate the divergence of the position error through integration.

For the SINS/GPS loosely coupled KF-based navigation system, the system fusion the GPS position information and position, velocity and attitude information computed by only SINS, as Equations (A7) to (A9) in Appendix A show. The position information computed by only SINS diverges over time. The GPS position computing is without integral, so that GPS position information has almost no drift. Thus, based on KF, GPS position information can restrain the divergence of only SINS.

However, as Figure 2 shows, the IMU data are directly fused with GPS position information, without an inertial computer. Thus, there is no accumulation of error due to integration in the proposed method. As Equation (6) shows, the relationship between LSTM input information and LSTM output information is linear. The linear parameters are $W_{O}$ and $b_{C}$. For a trained LSTM net, the structural parameters are constant. The error of LSTM estimation position is dependent on the parameters. The LSTM structure determines that the error of LSTM estimation position is linear. It is well known that suppression of linear error is easier than suppression of nonlinear error. Therefore, the LSTM position estimation method, which only produces linear error, makes the application of inertia information more convenient.

Both the KF method and the LSTM method can fuse GPS and an inertial sensor to restrain the error divergence of only SINS. There are some differences between the KF method and the LSTM method. For the KF information fusion method, it is a “white box.” It is applicable to the same navigation systems with the same system equation and same measurement equation. The system function and fusion function are definite. The system noise and measurement noise must be Gaussian white noise. With the same initial value, the position computed by the KF method is the same in every time. For the LSTM information fusion method, it is a “black box.” It is not required to give the specific equations of the system. The trained LSTM method model is only applicable to the system that provides the training and testing data. The LSTM output is based on the gate threshold value and the training cost function. There are no specific requirements for system noise or measurement noise. With the LSTM information fusion method, the position estimation output is different even with the same hyperparameters in every time. The outputs are distributed in an accuracy range.

## 6. Conclusions

In this study, we proposed a method using an LSTM net to estimate position information based on IMU data and GPS position information, because of its “memory” layer. Simulation and experimental results have shown that the proposed method is effective. The range of LSTM hyperparameters is explored through simulations. The LSTM information fusion method can restrain the position divergence of only SINS. In simulations, the LSTM method computed position error STD is 52.38% of only SINSs. The biggest simulation radial error estimated by the LSTM method is 0.57 m. In the experiments, the LSTM method computed position error STD at 23.08% for only SINSs. The biggest experimental radial error the LSTM method estimated was 1.31 m.

The trained LSTM fusion model is not applicable to all IMUs. It is only applicable to the IMU which provides the training data. The LSTM fusion method is used to estimate position information based on IMU data and GPS position, which only produces linear error, which makes the subsequent application of navigation information more convenient.

All in all, the trained LSTM is a dependable fusion method for combining IMU data and GPS position information to estimate position.

## Data Availability

The data presented in this study are available on request from the corresponding author. The data are not publicly available due to ethics.

## Appendix A

IMU data are computed into positioning/navigation information by a SINS algorithm [1]. The pose, velocity, and position information updating functions are shown below:

(A1) $$\dot{C_{b}^{n}}=C_{b}^{n}\left(\omega_{ib}^{b}\times \right)-\left(\omega_{in}^{n}\times \right)C_{b}^{n},$$

where $\omega_{ib}^{b}$ is the IMU measurement angular velocity and $\omega_{in}^{n}$ is the rotation of navigation coordinate relative to the inertial coordinates.

(A2) $$\dot{v^{n}}=C_{b}^{n}f_{sb}^{b}-\left(2\omega_{ie}^{n}+\omega_{en}^{n}\right)\times v^{n}+g^{n},$$

where $\dot{v^{n}}$ is the geometric acceleration of the vehicle in the navigation coordinate system. $f_{sb}^{b}$ is the IMU measurement accelerated velocity, while$\;g^{n}$ is the equatorial gravitation. $-\left(2\omega_{ie}^{n}+\omega_{en}^{n}\right)\times v^{n}$ is the harmful acceleration. Equation (A2) is called the inertial navigation specific force equation. We can get the velocity information by integrating $\dot{v^{n}}$ once, and the position information by integrating $\dot{v^{n}}$ twice. This is the base equation of the SINS algorithm:

(A3) $$\dot{p}=M_{pv}v^{n},$$

where $M_{pv}=\left[\begin{array}{ccc} 0 & 1/R_{Mh} & 0\\ \sec L/R_{Nh} & 0 & 0\\ 0 & 0 & 1 \end{array}\right]$.

In the navigation coordinates, the position error equations in dynamic conditions are as follows:

(A4) $$\dot{\phi }=M_{aa}\phi +M_{av}{\delta v}^{n}+M_{ap}\delta p-C_{b}^{n}\varepsilon^{b},$$

where ${\;M}_{aa}=-\left(\omega_{in}^{n}\times \right)=\left[\begin{array}{ccc} 0 & \omega_{U}+v_{E}\tan L/R_{Nh} & -\omega_{N}-v_{E}/R_{Nh}\\ -\omega_{U}-v_{E}\tan L/R_{Nh} & 0 & -v_{E}/R_{Nh}\\ \omega_{N}+v_{E}/R_{Nh} & v_{N}/R_{Mh} & 0 \end{array}\right]$, $M_{av}=\left[\begin{array}{ccc} 0 & -1/R_{Mh} & 0\\ 1/R_{Nh} & 0 & 0\\ \tan L/R_{Nh} & 0 & 0 \end{array}\right],{\;M}_{ap}=\left[\begin{array}{ccc} 0 & 0 & v_{N}/R_{Mh}^{2}\\ -\omega_{ie}\sin L & 0 & -v_{E}/R_{Nh}^{2}\\ \omega_{ie}\cos L+v_{E}\sec^{2}L/R_{Nh} & 0 & -v_{E}\tan L/R_{Nh}^{2} \end{array}\right],$ where L, λ, and h denote the local latitude, longitude, and height, respectively; v defines the body velocity vector; ϕ describes the body pose errors; ε is the constant gyroscope drifts; the subscripts E, N and U denote the projection on the east, north, and up axes of the local coordinates, respectively. $R_{Nh}=R_{N}+h$, $R_{Mh}=R_{M}+h$, $R_{N}=R_{e}/\sqrt{1-e^{2}\sin^{2}L}$, and $R_{M}=R_{e}\left(1-e^{2}\right)/{\left(1-e^{2}\sin^{2}L\right)}^{3/2}$, where $R_{e}$ is the ellipse major axis and e is the ellipse eccentricity.

The velocity error equations in the dynamic condition are as follows:

(A5) $$\delta {\dot{v}}^{n}=M_{va}\phi +M_{vv}{\delta v}^{n}+M_{vp}\delta p+C_{b}^{n}\nabla^{b},$$

where $M_{vp}=\left[\begin{array}{ccc} 2\left(v_{N}\omega_{N}+v_{U}\omega_{U}\right)+v_{E}v_{N}\sec^{2}L/R_{Nh} & 0 & v_{E}\left(v_{U}-v_{N}\tan L\right)/R_{Nh}^{2}\\ -v_{E}\left(2\omega_{N}+v_{E}\sec^{2}L/R_{Nh}\right) & 0 & v_{E}v_{N}/R_{Mh}^{2}+v_{E}^{2}\tan L/R_{Nh}^{2}\\ 2\omega_{U}v_{E}+g_{e}\sin2L\left(\beta -4\beta_{1}\cos2L\right) & 0 & -v_{E}^{2}/R_{Mh}^{2}-v_{N}^{2}/R_{Nh}^{2}+\beta_{2} \end{array}\right],$

$$\begin{array}{l} \;M_{vv}=\left[\begin{array}{ccc} \left(v_{N}\tan L-v_{U}\right)/R_{Nh} & 2\omega_{U}+v_{E}\tan L/R_{Nh} & -2\omega_{N}-v_{E}/R_{Nh}\\ -2\omega_{U}-2v_{E}\tan L/R_{Nh} & -v_{U}/R_{Mh} & -v_{N}/R_{Mh}\\ 2\omega_{N}+2v_{E}/R_{Nh} & 2v_{N}/R_{Mh} & 0 \end{array}\right],\\ M_{va}=\left[\begin{array}{ccc} 0 & -f_{U} & f_{N}\\ f_{U} & 0 & -f_{E}\\ -f_{N} & f_{E} & 0 \end{array}\right], \end{array}$$

where ∇ denotes the accelerometer zero biases, f is the specific force, and β is the gravitation flattening, with β≈1/188.6, $\beta_{1}\approx 1/8\left(2\beta f+f^{2}\right),\;\mathrm{and}\;\beta_{2}\approx 3.08\times 10^{-6}s^{-2}$.

The position error equations in the dynamic condition are as follows:

(A6) $$\delta \dot{p}=M_{pv}{\delta v}^{n}+M_{pp}\delta p,$$

where $M_{pp}=\left[\begin{array}{ccc} 0 & 1/R_{Mh} & -v_{N}/R_{Mh}^{2}\\ v_{E}\sec L\tan L/R_{Nh} & 0 & -v_{E}\sec L/R_{Nh}^{2}\\ 0 & 0 & 0 \end{array}\right].$

SINS information and GPS information can be fused by a KF [28]. For the loosely coupled method, the state vector is $X={\left[\delta p,{\delta v}^{n},\phi ,\nabla^{b},\varepsilon^{b}\right]}^{T}$ and the system equation is

(A7) $$\dot{X}=FX+GW,$$

where, F is the system matrix, G is the system noise matrix, and W is the system noise.

(A8) $$F=\left[\begin{array}{cc} \begin{array}{ccc} M_{pp} & M_{pv} & 0_{3\times 3}\\ M_{vp} & M_{vv} & M_{va} \end{array} & \begin{array}{cc} 0_{3\times 3} & 0_{3\times 3}\\ C_{b}^{n} & 0_{3\times 3} \end{array}\\ \begin{array}{ccc} M_{ap} & M_{av} & M_{aa}\\ 0_{3\times 3} & 0_{3\times 3} & 0_{3\times 3}\\ 0_{3\times 3} & 0_{3\times 3} & 0_{3\times 3} \end{array} & \begin{array}{cc} 0_{3\times 3} & -C_{b}^{n}\\ 0_{3\times 3} & 0_{3\times 3}\\ 0_{3\times 3} & 0_{3\times 3} \end{array} \end{array}\right],\;G=\left[\begin{array}{c} \begin{array}{cc} 0_{3\times 3} & 0_{3\times 3}\\ 0_{3\times 3} & 0_{3\times 3} \end{array}\\ \begin{array}{cc} 0_{3\times 3} & 0_{3\times 3}\\ I_{3\times 3} & 0_{3\times 3}\\ 0_{3\times 3} & I_{3\times 3} \end{array} \end{array}\right],\;W=\left[\begin{array}{c} w_{g}\\ w_{a} \end{array}\right],$$

The measurement vector is

(A9) $$Z=\left[\begin{array}{c} \left(L_{sins}-L_{GPS}\right)R\\ \left(\lambda_{sins}-\lambda_{GPS}\right)RcosL \end{array}\right]=\left[\begin{array}{c} R\delta L+N_{n}\\ RcosL\delta \lambda +N_{e} \end{array}\right]=HX+V,$$

where, H is the system measurement matrix, $V={\left[\begin{array}{cc} N_{n} & N_{e} \end{array}\right]}^{T}$ is the measurement noise, and $N_{n}$ and$\;N_{e}$ are the GPS measurement noise of North position and East position, respectively.

(A10) $$H=\left[\begin{array}{cc} \begin{array}{cc} R & 0\\ 0 & RcosL \end{array} & 0_{13\times 2} \end{array}\right].$$

The KF includes a one-step state prediction equation, a state estimation equation, a filtering gain equation, a one-step prediction mean square error equation, and an estimated mean square error equation. They are listed below:

(A11) $${\hat{X}}_{k+1,k}=XF_{k+1,k}{\hat{X}}_{k}$$

(A12) $${\hat{X}}_{k+1}={\hat{X}}_{k+1,k}+K_{k}\left(Z_{k}-H_{k}{\hat{X}}_{k+1,k}\right)$$

(A13) $$K_{k+1}=P_{k+1,k}H_{k}^{T}{\left(H_{k}P_{k+1,k}H_{k}^{T}+R_{k}\right)}^{-1}$$

(A14) $$P_{k+1,k}=F_{k+1,k}P_{k}F_{k+1,k}^{T}+G_{k+1,k}Q_{k}G_{k+1,1}^{T}$$

(A15) $$P_{k+1}=\left(I-K_{K}H_{K}\right)P_{k}{\left(I-K_{K}H_{K}\right)}^{T}+K_{k}R_{k}K_{k}^{T},$$

where, ${\hat{X}}_{k+1,k}$ denotes the prediction of the state vector from $t_{k}$ to $t_{k+1}$, ${\hat{X}}_{k}\;$denotes the prediction of the state vector at $t_{k}$, $K_{k}$ denotes filtering gain matrix, $P_{k+1,k}$ denotes one-step estimated mean square error matrix from $t_{k}$ to $t_{k+1}$, $P_{k}$ denotes estiated mean square error matrix, $Q_{k}$ is system noise covariance matrix, and $R_{k}$ denotes measurement noise covariance matrix.
